# Supplementary figures and images for: A CENH3 mutation promotes meiotic exit and restores fertility in SMG7-deficient Arabidopsis
Source: PLoS Genet. 2021 Sep 30;17(9):e1009779. doi: 10.1371/journal.pgen.1009779 (PMC8509889; doi:10.1371/journal.pgen.1009779)

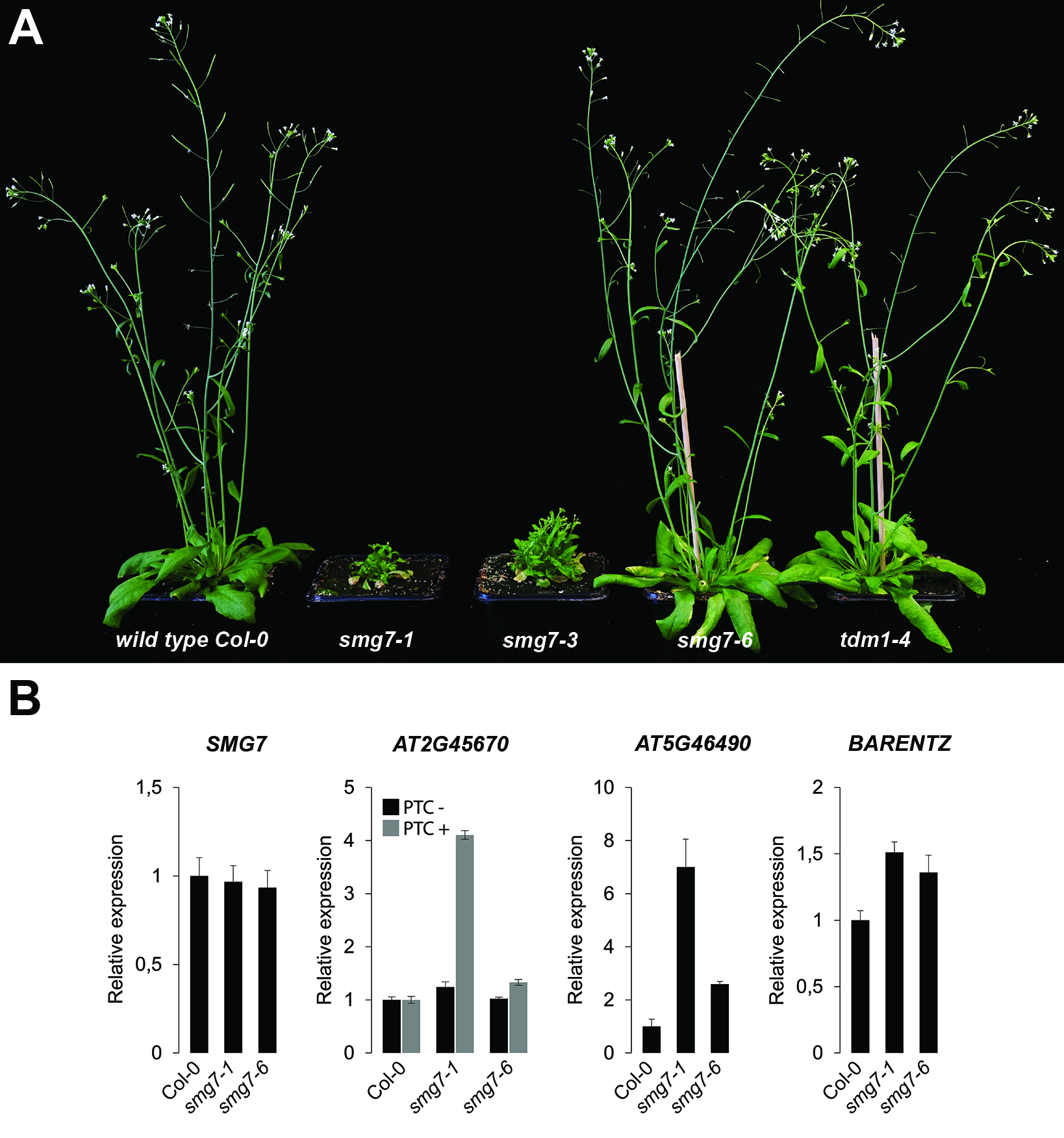

Supplement: S1 Fig — (A) Five week-old Arabidopsis mutants homozygous for the indicated alleles. (B) Quantitative RT-PCR analysis of SMG7 mRNA from the region located upstream of the T-DNA insertions and of transcripts targeted by NMD in smg7-1 and smg7-6 mutants. Two mRNA splice variants, one containing a premature termination codon (PTC+), were quantified for the AT2G45670 locus. Error bars indicate standard deviations from three biological replicas. (TIF) [file pgen.1009779.s008.tif]

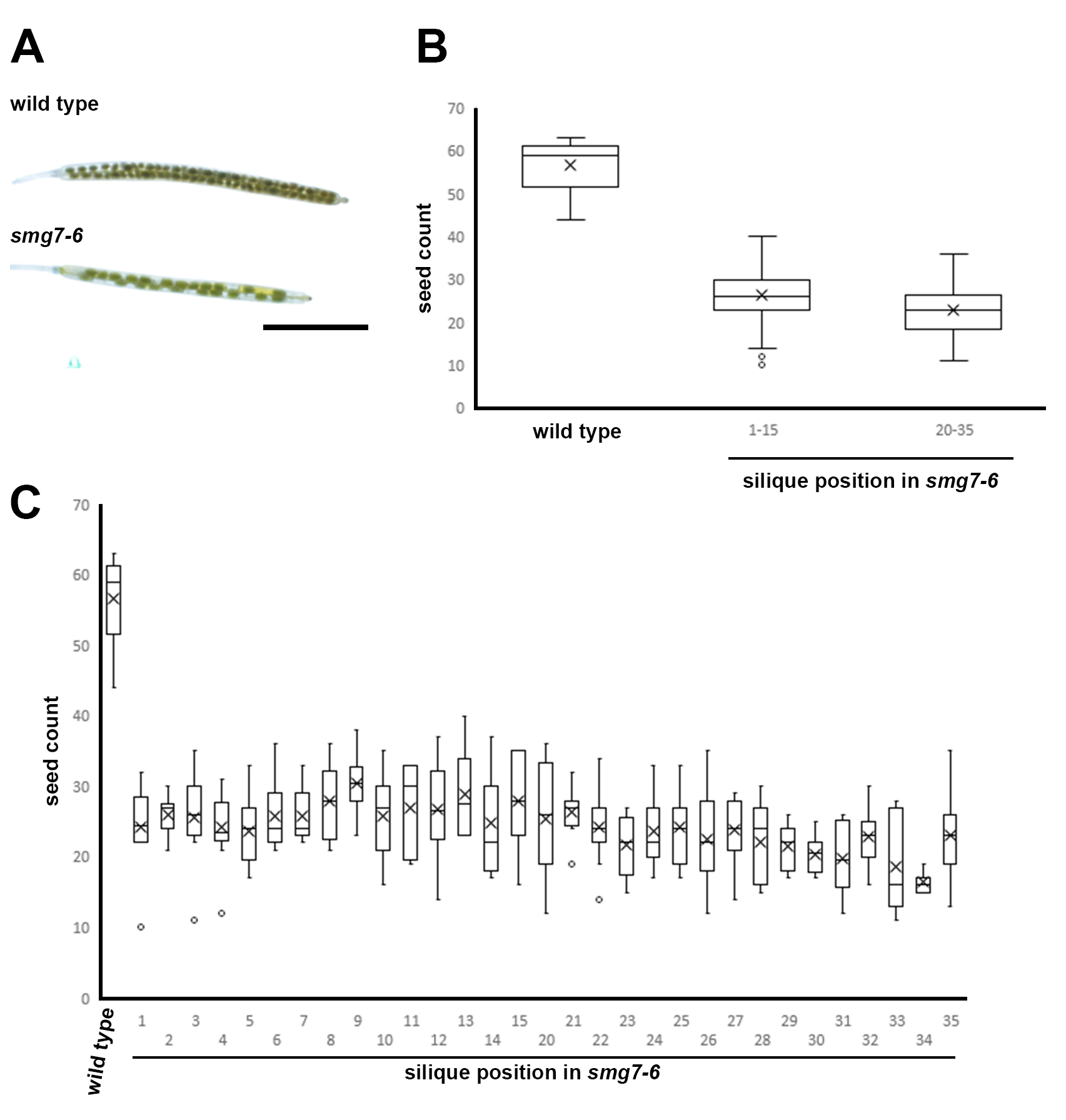

Supplement: S2 Fig — (A) Siliques from wild type and smg7-6 plants produced by pollination with wild type pollen. Scale bar = 0.5 cm. (B) Box-plot diagrams showing quantification of seed count per silique from wild type (N = 14) and smg7-6 plants pollinated with wild type pollen. Yield from siliques at positions 1 to 15 (N = 136) and 20 to 35 (N = 153) is indicated for smg7-6 mutants. (C) Box-plot diagram showing quantification of seeds in siliques at indicating positions along the main inflorescence bolt with 1 indicating the lowest position. 5 to 14 siliques were counted per position. (TIF) [file pgen.1009779.s009.tif]

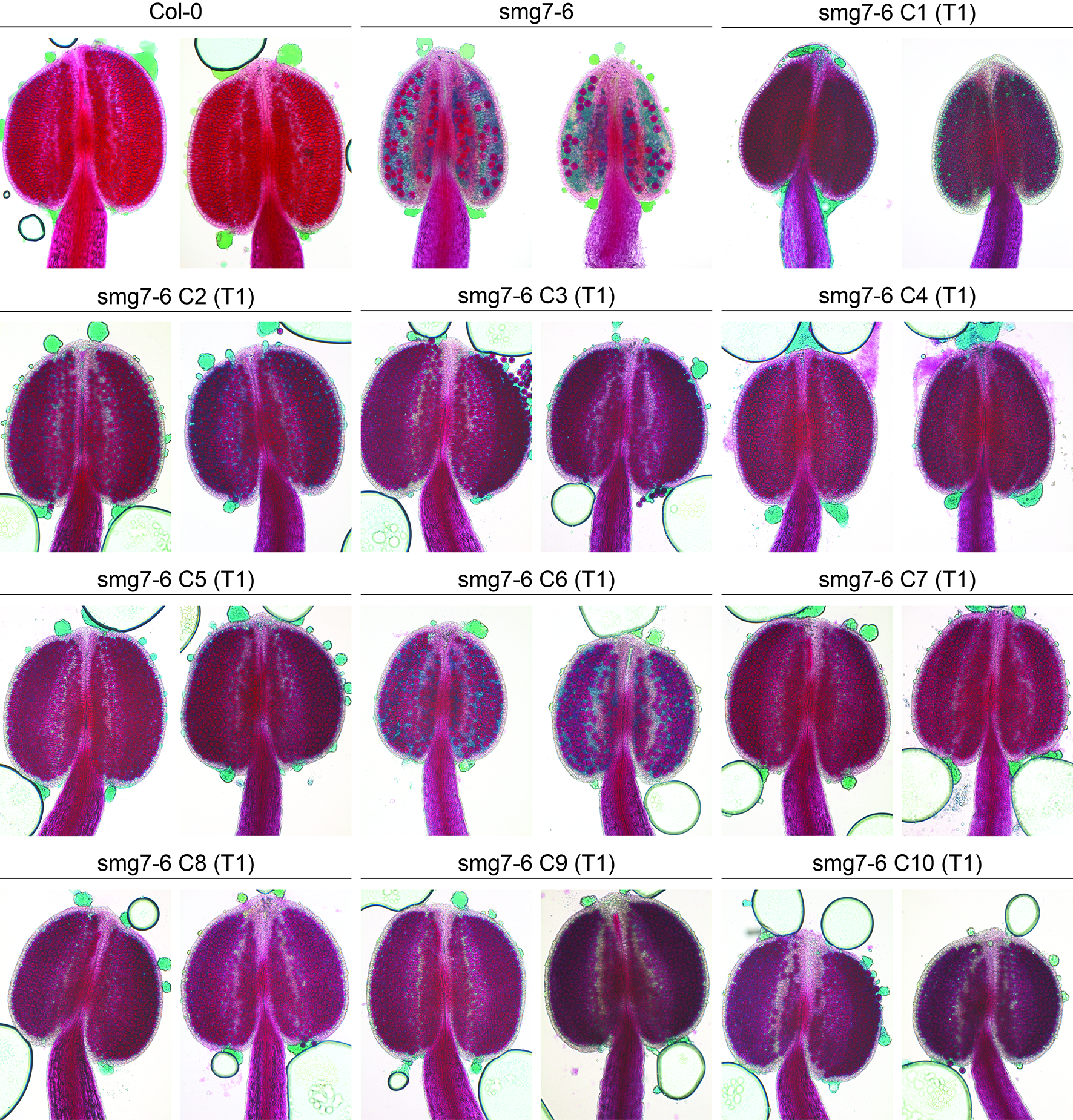

Supplement: S3 Fig — Anthers from 10 independent T1 smg7-6 transformants carrying the pSMG7::SMG7 construct with viable pollen detected by Alexander staining. All T1 lines show a restoration of viable pollen. (TIF) [file pgen.1009779.s010.tif]

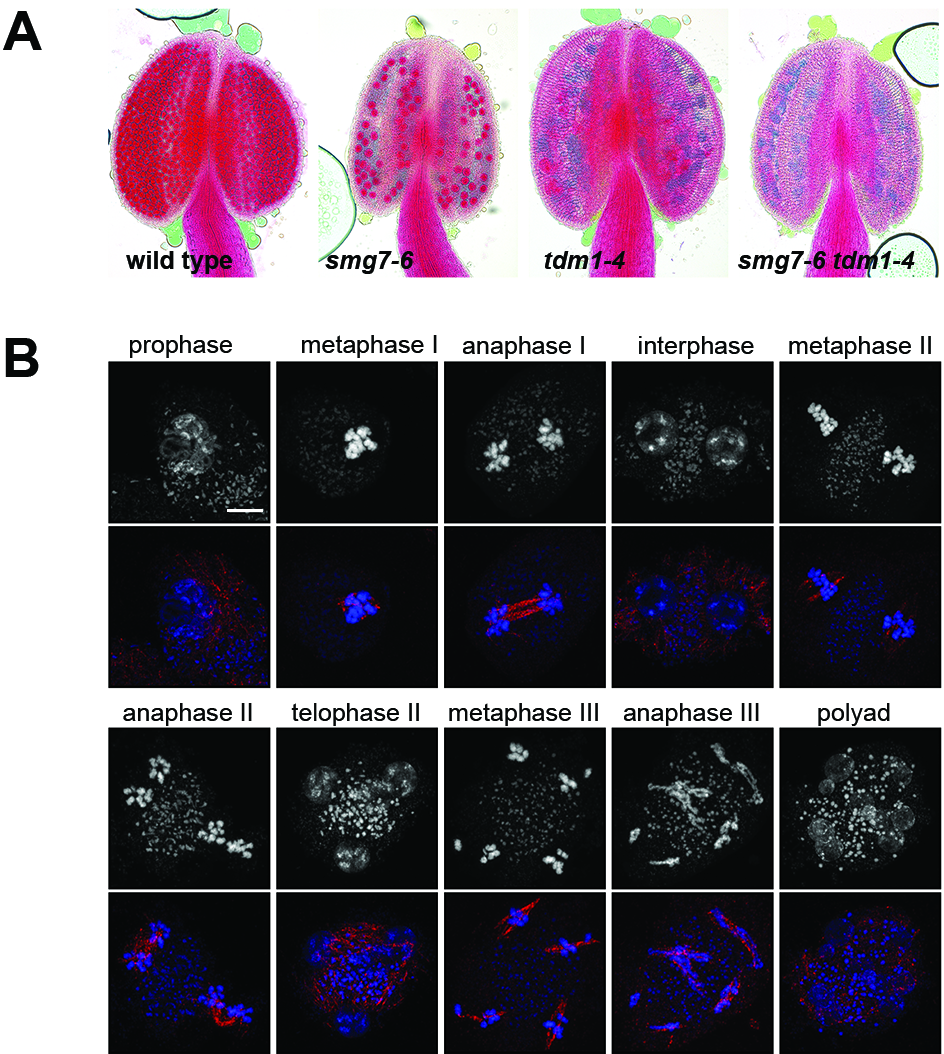

Supplement: S4 Fig — (A) Anthers of the indicated mutants after Alexander staining. Viable pollen stain red. (B) Meiotic progression in PMCs of smg7-6 tdm1-4 double mutants. Spindles are stained with anti-α-tubulin antibody (red), DNA is counterstained with DAPI. Scale bar corresponds to 5 μm. (TIF) [file pgen.1009779.s011.tif]

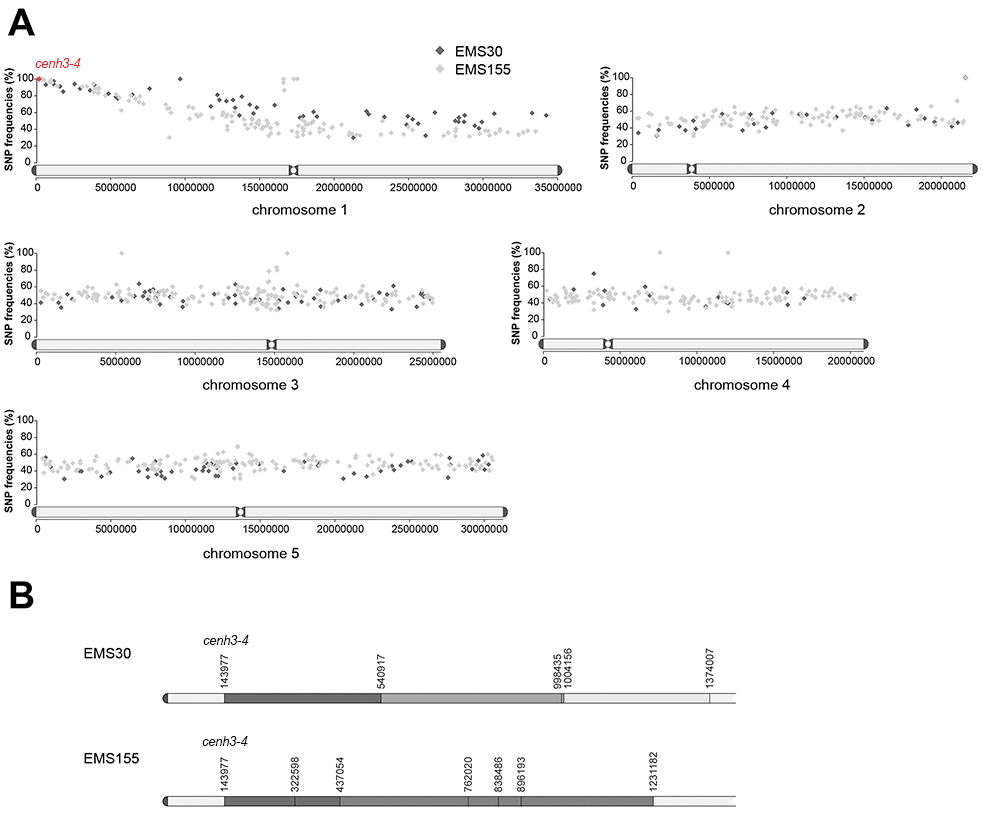

Supplement: S5 Fig — (A) Genome-wide distribution of de novo mutations and their frequency in fertile B2plants generated from backcrosses of EMS30 and EMS155 lines with parental smg7-6 plants. (B) De novo mutations at the left arm of chromosome 1 in the EMS30 and EMS155 lines. Coordinates of these mutations in TAIR10 annotation are indicated. (TIF) [file pgen.1009779.s012.tif]

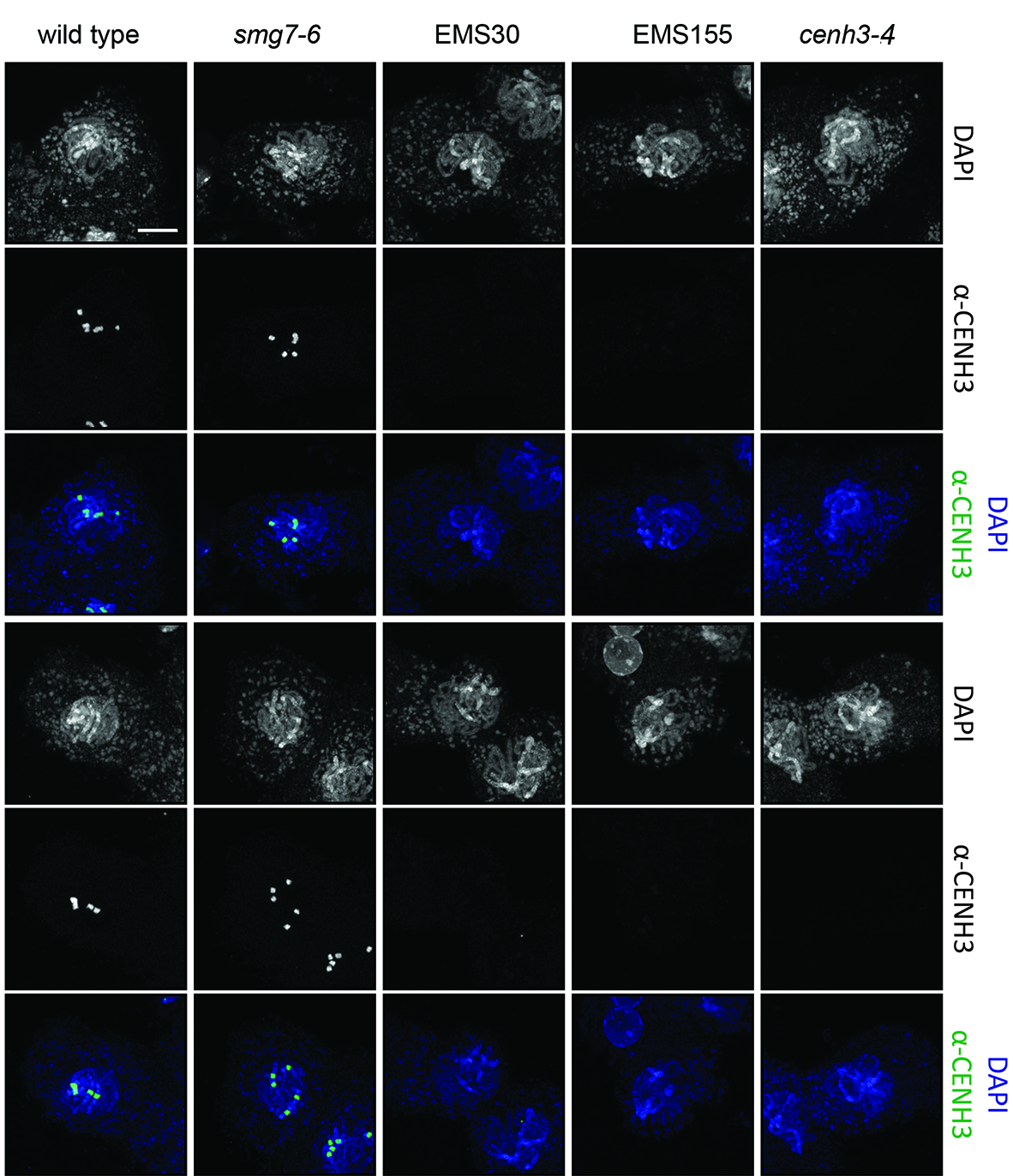

Supplement: S6 Fig — CENH3 is visualized with CENH3 antibody; DNA is counterstained with DAPI (blue). Scale bar represents 5 μm. (TIF) [file pgen.1009779.s013.tif]

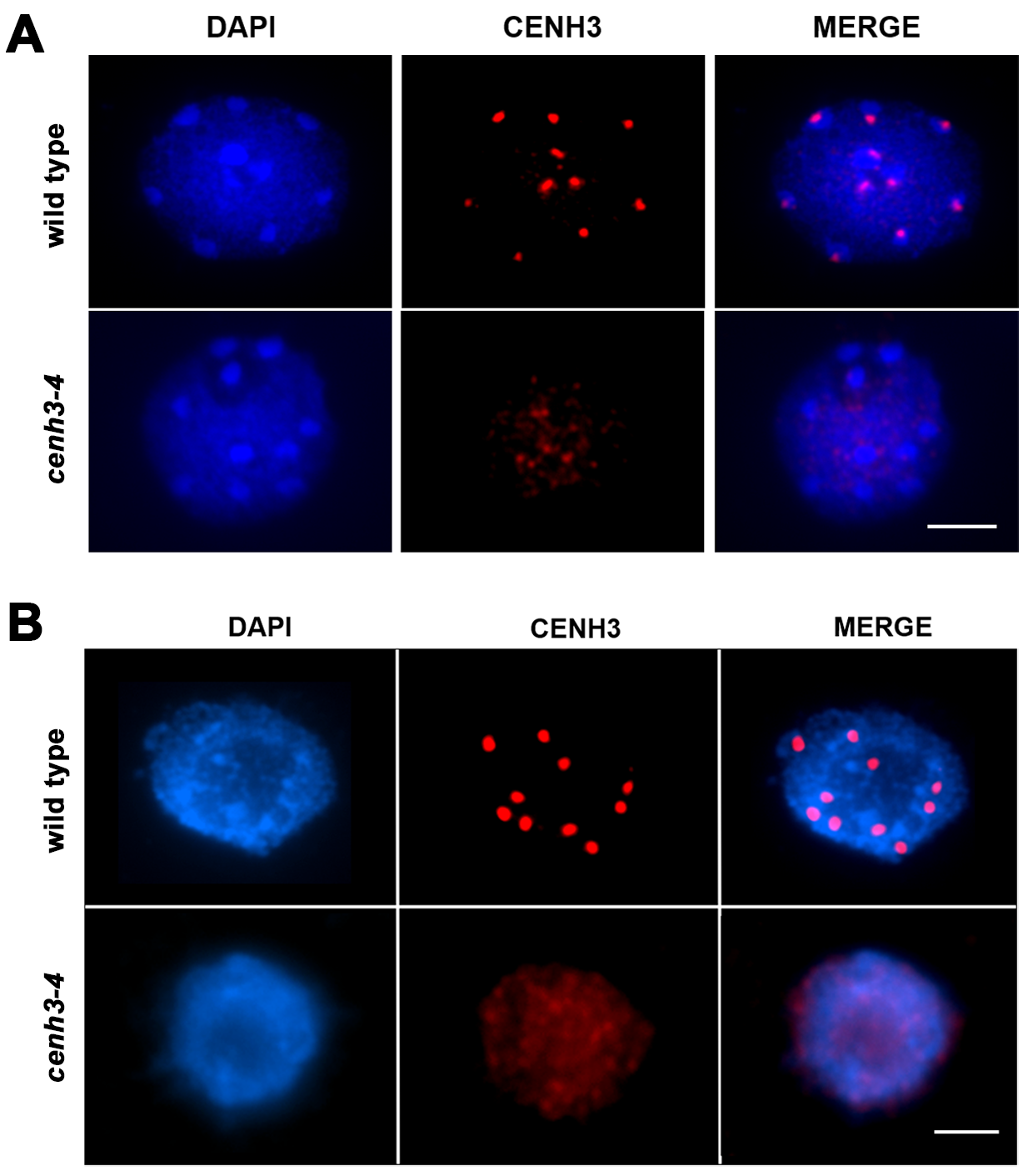

Supplement: S7 Fig — CENH3 is visualized with CENH3 antibody; DNA is counterstained with DAPI (blue). Scale bar represents 5 μm. (TIF) [file pgen.1009779.s014.tif]

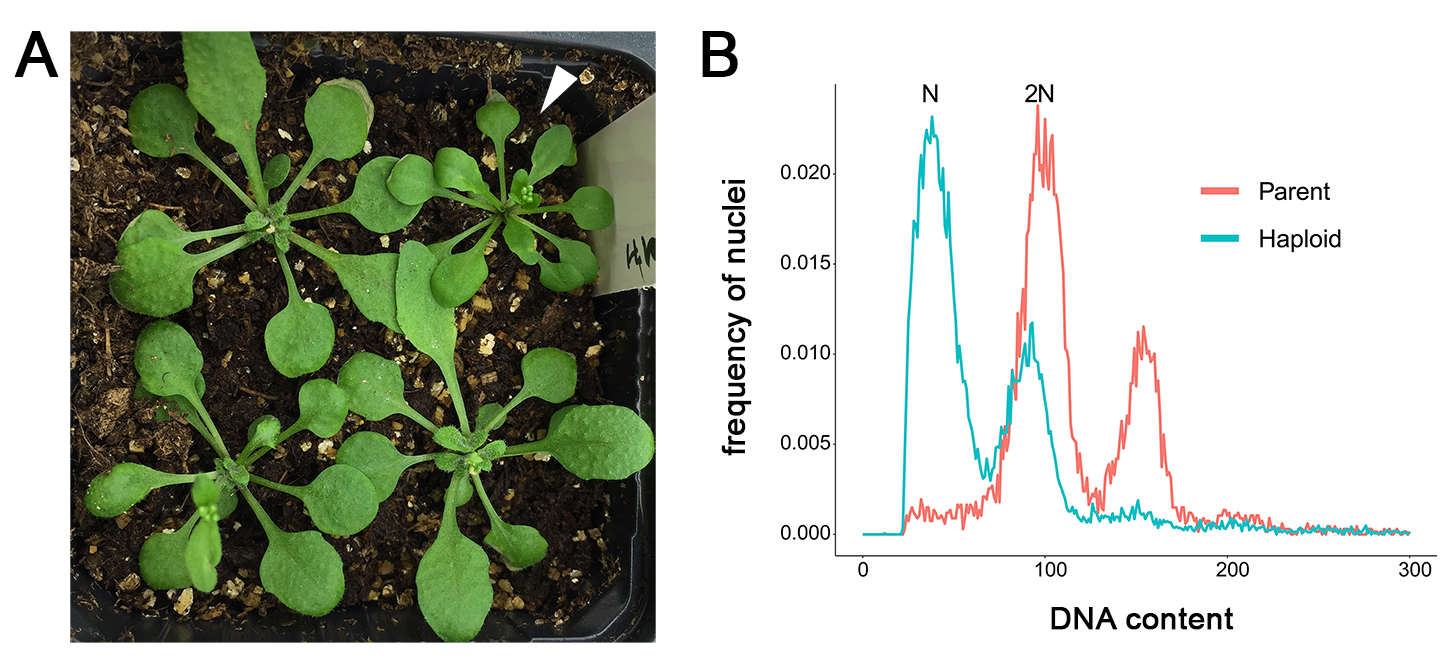

Supplement: S8 Fig — (A) The haploid plant (indicated by arrowhead) was recognized based on its trichomeless phenotype. (B) Nuclear content of inflorescence nuclei from the haploid and a parent plant determined by flow cytometry. (TIF) [file pgen.1009779.s015.tif]
