# Supplementary material for: A CENH3 mutation promotes meiotic exit and restores fertility in SMG7-deficient Arabidopsis
Source: PLoS Genet. 2021 Sep 30;17(9):e1009779. doi: 10.1371/journal.pgen.1009779 (PMC8509889; doi:10.1371/journal.pgen.1009779)
Supplement: Attachment — Submitted filename: Response to Reviewers.docx [file pgen.1009779.s018.docx]

Reviewer's Responses to Questions

I want to thank all reviewers for positive evaluation and constructive comments on our work. Here is our detailed point-by-point response to reviewer´s critique an suggestions.

Reviewer #1: 

Minor point:

Line 281. The chromosome mentioned in citation 51 does indeed have only 110 kb of canonical centromere sequences. This work was performed before the realization of the epigenetic nature of plant centromeres became known. Subsequent work on this chromosome (Liu et al 2015, PNAS 112: E1263-1271) found that it actually has a de novo centromere elsewhere and the canonical site is not active. The de novo centromere has a CENH3 domain of 288 kb, so that chromosome does indeed have a small centromere. Thus, the point of the authors is still correct. Because this is not a major consideration for the manuscript as a whole, the authors might consider citing instead Jin et al., 2005, Plant Cell 17: 1412-1423. The same chromosome was analyzed in that paper as well and that was also before the realization of epigenetic aspects of plant centromeres. However, a number of small centromeres were examined in this latter manuscript and the amount of CENH3 was semi quantified. Those centromeres all work albeit some have reduced transmission.

Thank you for pointing it out. We have corrected the sentence and updated the reference.

Reviewer #2: 

Major concerns:
1. The suppressor screen used in the study is based on the infertility of the first 20-25 flowers (later flowers are partially fertile) in the smg7-6 mutant. However, the authors admit that pollen production shows a similar reduction in all flowers. The authors suggest that this is due to " unknown physiological factors that affect pollination or fertilization" (line 155). However, differences in the course of male and female meiosis in this mutant are also possible. Pollination influencing factors can come from the stigma or the pollen itself. Therefore, I suggest carrying out a simple experiment with the analysis of seed setting in siliques pollinated with pollen from wild plants and smg7-6 in the first 20 and later flowers.

We now provide this analysis in new Supplementary Figure S2. Interestingly, we noticed reduced ability of *smg7-6* plants to produce seeds when pollinated with wild type pollen suggesting that the *smg7-6* mutation also affects female meiosis. Nevertheless, this experiment did not uncover substantial difference between female fertility of early and late flower. This strengthens the conclusion that difference in seed production is likely due to physiology of pollination or fertilization, rather than an effect on meiosis.

2. It is not understandable for me why the cenh3-2 mutant does not show any more serious developmental disorders. Decreasing the CENH3 level to the level of 20-30% causes significant phenotypic effects (Lermontova et al. Plant J. 2011), while the authors indicate that the protein level in cenh3-2 corresponds to about 10% of the protein in wild type (and this is confirmed in RT-PCR of which corresponds to the level of the transcript). The authors should check the expression level more carefully by analysing it in vegetative tissues such as rosette leaves (at transcript and/or protein level).

Indeed, this is quite surprising observation. We have checked by immunostaining presence of CENH3 signal in leaves and root tips (new Supplementary Figure 7) and we see substantial reduction of the signal similar to data we present in the main manuscript on pachytene and tapetum cells. I should also mention that our attempts to combine *cenh3-4* allele with the null *cenh3-1* allele failed suggesting that *cenh3-4* mutants have the least amount of CENH3 sufficient to support cell viability.

3. In connection with the previous point, it seems necessary to check whether a reduction of CENH3 expression (e.g. in RNAi lines) will also suppress the smg7-6 phenotype. Alternatively, suppression can be checked for other weak CENH3 mutant lines. I assume this team has access to such mutants.

We have not performed this experiment. I am of the opinion that using RNAi technology brings a number of caveats. RNAi can affect RNA stability, but can also hinder translation. The RNAi lines were characterized for the effect on the CENH3 mRNA (20-30%), but not the protein level. Thus, it is possible that the extend of CENH3 suppression on protein level is greater than reported. In addition, efficiency of RNAi technology may vary between tissues and cell types. Hence, crossing RNAi lines with *smg7-6* may go either way, but unless we perform very deep characterization of the RNAi lines, this experiment will not allow any firm conclusions, particularly in respect to the meiotic phenotype. As for CENH3 point mutations, there are only three other ones described in Arabidopsis (see also comments of reviewer 3). Two of them are null and, therefore, lethal, while the remaining one is A86V substitution that gives relatively weak haploid induction phenotype. Besides haploid induction, this mutant has not been characterized and it is unknown, whether it affects CENH3 level. While it would be interesting to analyze this mutant, we have not asked Ann Britt for sending us the seeds.

4. Analysis of CENP-C and KNL2 proteins is a nice extension of the proposed hypothesis, however microscope images can hardly be considered as quantitative. Authors should mitigate the conclusions unless they present alternative quantitative analysis of the levels of these proteins bound to centromeres in the cenh3-2 mutant.

Indeed, the pictures presented in the manuscript were not of a good quality. Now, we provide new Figure 8, which clearly shows substantial reduction of kinetochore proteins in *cenh3-4* mutants. In addition, we include analysis of another kinetochore protein, MIS12. We also attempted to quantify the difference in signals, but this cannot be done rigorously. Due to very weak signals in *cenh3-4*, different exposure times are needed for imaging wild type and *cenh3-4* mutants. The signals in *cenh3-4* are not readily detectable under conditions used for imaging wild type nuclei. Thus, the obtained signal quantities are not directly comparable. For your information, we include Figure 1R which shows quantification of kinetochore proteins on centromeres in wild type and *cenh3-4* mutants. The difference is still obvious despite the different conditions used for image acquisition that overestimate signals in *cenh3-4* plants due to longer exposure times. Because of this technical caveat, we refrain to include this data in the manuscript. We believe that data presented in the new Figure 8 are sufficient to show substantial reduction of kinetochore proteins on *cenh3-4* centromeres.

Figure 1R. Quantification of kinetochore proteins on centromeres by immunocytology. 50 to 70 root nuclei were analyzed for each data point.

5. In line 293 the authors present the hypothesis that smg7-6 phenotype suppression in cenh3-2 is caused by inefficient formation of centromere-microtubule interactions which hinder spindle reassembly. To confirm this hypothesis, it should be enough to reduce the amount of one of the kinetochore proteins, eg KNL2, and see if it also allows for suppression. Can the authors perform such an experiment?

We generated *smg7-6 knl2* double mutants. However, these plants showed even greater reduction of fertility than *smg7-6* plants. In our hands, already *knl2* plants show reduced fertility and vegetative growth defects indicating a broader role of the protein in Arabidopsis.


Minor points:
Line 125: Please specify what genotype the description in this line refers to.
Line 182: Please specify: was the double suppressor carrying the two independent suppressor mutations? Please rephrase.
Fig. 6: Could you please add colours, especially to fig. B, so the figure is more readable?

Corrected according to the reviewer´s suggestions.

Reviewer #3: 

Major points
The smg7-6 phenotype is complex and incompletely characterized. This is not a mortal flaw, but it complicates the analysis of the reduced CENH3 effect. By the way, the reported 1:3 live:dead ratio is unexpected. The figure seems to suggest that this is a frequent event. It needs statistical quantification: are there ever 2:2 events? The 1 viable: 3 dead pollen trait in the tetrad could be due to one spore capturing 5+n chromosomes and depleting the others to be 5-n. Alternatively, the live spore could be the only one that has 5 different chromosomes. Does the progeny display aneuploidy?

We now include new panel 1D with the quantification of tetrads with viable pollen in *smg7-6 qrt* mutants. Furthermore, we have not detected any aneuploidy in smg7-6 plants. We now discuss this observation in the result section (lines 151 to 156).

It makes sense to number the cenh3 mutants progressively with publication. Kuppu (2015) described a cenh3 mutant (A86V), although they did not number it. The cited biorXiv paper by Marimuthu [60] describes a knock-out mutation in CENH3 and calls it 3-3. This should make the mutation described in this manuscript cenh3-4, or alternatively 3-3 if that is 3.-4.

Thanks for pointing it out. We discovered the *cenh3* allele before these papers were published, therefore we called it *cenh3-2* allele, but the name should be updated according to the current state of the knowledge. Therefore, we renamed the allele to *cenh3-4* as suggested.

L212. The reduced root growth trait could be caused by independent linked mutations. Has the trait been confirmed for both independent instances of the cenh3-2 allele, or for the transheterozygote (cross of two mutants)? Alternatively, it should be complemented by transformation with a wild-type CENH3.

We include new data in Figure 9B showing that both independent suppressor lines carrying the *cenh3-4* mutation exhibit the slow growth root phenotype.

The delayed timing explanation for suppression of smg7-6 by cenh3-2 could be better supported if analyzed as shown in Fig.3B,C.

Indeed, such analysis may provide further insights on how CENH3 deficiency rescues *smg7-6* phenotype. However, this is rather demanding technology in terms of men power and microscopy time, and I must say that we have not given sufficient priority to this mutant and have not generated required reporter lines. Our major motivation to set up the suppressor screen was to identify genes involved in meiotic exit and transition to gametophytic development. While the *cenh3* mutation we identified in the screen represents interesting resource for analysis of centromere biology, it is likely not directly linked to the mechanism that governs meiotic exit. Its rescue effect is rather indirect through affecting chromosome segregation/cell cycle progression. For that reason, we rather prioritize other suppressor mutations which may be more informative in terms of mechanistic understanding of the meiotic exit in Arabidopsis.

I wonder if another explanation for the suppression of smg7-6 is that cenh3-2 centromeres capture fewer spindle fibers and are less likely to be missegregated to a wrong pole or cell.

This is very interesting thought that may be testable using superresolution microscopy.

Fig.6. I found the figure unclear. In A, the splice products of wild type and mutant should be described. Have the cDNA’s been sequenced and is the structure of the resulting CENH3 the same as in the wild type? In B, the log relative expression is useful when comparing regulation, but in this case it is not clear, particularly because the text in Results uses a fold estimate. Why not use % mRNA standardized on WT? Why are both mutant and wild-type spliced forms measured in B? Spliced-CENH3-A. Why is a log used in B and not in D and E?

Indeed, the *cenh3-4* mutation results in two splice forms, one fully spliced and one retaining the intron 3. We updated the Figure 6A to provide more thorough information on the sequence features surrounding the mutation site. In order to detect fully spliced mRNA in wild type and *cenh3-4* plants, we had to design primer spanning the mutation site. To make sure that qRT-PCR amplification is not affected by the mutation, we performed the quantification with two sets of primers matching either the wild type or mutant allele. Both sets of primer gave similar result in both wild type and mutant samples indicating that this nucleotide change has negligible effect on primer performance. We updated text in the result section to clarify this point. As for the graphical representation of the results in Figure 6, both text and charts show fold change respective to wild type (we changed the labeling of the y axes to make this clearer). In chart 6B we plot log2 of fold change rather than fold change on linear scale as the fold-values span two orders of magnitude (appx 10 times lower amount of fully spliced mRNA and 10 time higher unspliced mRNA in CENH3 mutants) and this would be difficult to seen on a linear scale. In contrast, fold changes are very small in Fig 6D,E and linear scale is better for the visualization.

Figure 7 and Sup. FigS5. It would be helpful to quantify CENH3 the signals (Fig7 and Sup. FigS5)

We use different imaging conditions for wild type and cenh3-4 mutant plants, because the signal intensity is much lower in mutants. Thus, the signals cannot be directly compared. Please, se also our response to reviewer 2 on the detection of kinetochore proteins.

Figure 8: The KNL2 signals do not show 10 spots as expected in the wild-type. Why? For CENP-C, the cenh3-2 mutant there are more than 10? Why?

Data presented in Figure 8 were not of a good quality and some spots may represent background signal. This can happen in immunolabeling experiments, especially when signal is weak as in the case of *cenh3-4* mutants and longer exposure times are used. In such cases background signal becomes more prominent. We have now included new immunolocalization of the kinetochore proteins CENP-C, KNL2 and MIS12 in Figure 8 that show clear difference between wild type and mutant nuclei.

Discussion: Some may agree with the author’s conclusion that the results provide no support for the Dawe model of centromere size causing haploid induction. However, others may instead conclude that this paper supports such a model because haploid induction was demonstrated. I suppose “it depends” on the definition of haploid induction. To some, 0.2% is nearly background level. To others, it may be a significant signal. So, what is the background level? Is the control result significantly different from the reported rate?

Very good point. We have not performed extensive crossing experiment with wild type (I am not sure whether anybody published such data), so we cannot say what is the background level of haploid induction in wild type plants. Nevertheless, efficiency of haploid induction in *cenh3-4* plants is at least 10 times lower compared to the worst performing haploid inducing CENH3 mutations that have been published so far. Thus, taking in consideration a huge effect of the *cenh3-4* allele on centromere structure, efficiency of haploid mutation is surprisingly low.


Minor points
Abstract: the statement of no haploids is contradicted by the data in Results

Indeed, we corrected it.

L103: ...mutants, which contain a T-DNA…. domain, grow normally… Corrected.

L107 and other places: accepted usage is “wild-type” as single adjective (the wild-type strain), “wild type” as adjective + noun (the wild type grows tall). Corrected.
L136: metaphase I – telophase II, interkinesis, and metaphase II telophase II in wild type are measured as 46, 38, and 39 min: was this meant to be “metaphase I -to telophase I” or telophase II? If the latter applies, the timing does not make sense. Corrected.
L217: Prometaphase/metaphase duration in cenh3-2 is twice as WT yet the plant inflorescence displays normal growth: this needs an explanation.

In terms of duration of the entire cell cycle, which in rapidly dividing cells of the Arabidopsis root meristem lasts approximately 20 hrs, is 10 min delay in prometaphase rather negligible. Therefor, this defect does not necessarily need to result in a readily detectable growth phenotype of a developing organ. The fact that we see delay in root growth may indicate additional problems in *cenh3-4* mutants.

Table 1. Please define NEB. The legend states that “none of the examined cells….that entered the metaphase formed a metaphase plate”. How would one know that a cell entered metaphase if a metaphase plate was not formed?

It was supposed to be “…entered mitosis…”. We also expanded the legend to Table 1 to define NEB.

Fig.2B. Comparison to wild-type pictures should be added.

Wild type does not contain corresponding stages.

Fig.4. For clarity, it would be better to label the EMS30 and 155 lines with the genotype (smg7-6, cenh3-2). Figure 4B. It shows silique length, but no data on the female fertility (this is in the context of cenh3-2, mutant characterization).

We use this labeling on purpose to distinguish between the two independently obtained suppressor lines. We have not tested female fertility in suppressor lines by crosses to wild type. Until we performed the experiment presented in the new Supplementary Figure S2, we have considered *smg7-6* mutants to be female fully-fertile.

Fig.6. There is no doubt that this mutation results in a decreased amount of CENH3. It is not clear, however, how much lower. Is it 20, 10 or 5% of the wild-type amount? The western seems to indicate a quantity of CENH3 lower than that found in the ChIP. The assay and tissue is of course different, but one wonders what is the concentration.

We have not rigorously quantified CENH3 signal on Western blots. The *cenh3-4* signal in Figure 6C is roughly 50 times weaker than in wild type. There are at least two possible explanations for the discrepancy between the Western and ChIP data. First, loading of CENH3 on centromeric chromatin may be more efficient in *cenh3-4* mutants (or there is surplus of free CENH3 protein in wild type). Second, our ChiP method is based on pulling down larger chunks of fragmented chromatin with average size around 500 bp meaning that a single DNA fragment contains multiple nucleosomes. CENH3 in *cenh3-4* mutants may still occupy large centromeric domains and be just more diluted with canonical nucleosomes in comparison to wild type. Therefore, pulled-down DNA may not be linearly proportional to the amount of CENH3.

Fig.7. A normal young cell in the anther maternal tissue would be more informative than the tapetum, which is typically endopolyploid. Some of the panels in Fig.S5 would be useful to convey the effect of longer exposure time. Panel B did not show up in my download. I saw what could be the same panel in the authors biorXiv preprint.

We now include data showing reduction of CENH3 on *cenh3-4* centromeres in leaf and root nuclei. The panel 7B was indeed lost during last formatting of the manuscript, I apologize for that.

Fig.7. 180bp FISH coupled with CENH3 immuno (immuno-FISH) may be more complete.
Discussion: Worth mentioning: the centromere reduction may have a distinct deleterious effect on fitness in nature.

In fact, we have data suggesting altered response of *cenh3-4* mutants to stress conditions. We now pursue these preliminary data and this topic will be explored in our future research.

Method: did the ChIP analysis include a nuclease treatment? In other words, was it based on nucleosomes? This is an important detail for quantification and should be indicated in the method in addition to the reference [69].

Very good point. As explained in a previous paragraph, we used sonication and not nuclease treatment that would produce mononucleosomal fragments. We clarified it in the methods section.
